# Connecting Generations in the Digital Age: A Sentiment Analysis of Affective Bonds Between Senior Influencers and Young Audiences on a Chinese Danmaku Video Site

**DOI:** 10.1007/s10823-026-09598-5

**Published:** 2026-09-09

**Authors:** Yalun Wei, Keyu Zhang, Tianli Liu, Yong Hu

**Affiliations:** 1 Institute of Population Research, No. 186 Shuangfeng Road, Yubei District, 100190 Chongqing, China; 2 Department of Media and Communication, City University of Hong Kong, Hong Kong, 999077 Hong Kong SAR, China; 3 Institute of Population Research, Peking University, Yiheyuan Road, Haidian District, 100871 Beijing, China; 4 School of Journalism and Communication, Peking University, Yiheyuan Road, Haidian District, 100871 Beijing, China

**Keywords:** Senior digital influencer, Parasocial interaction, Active aging, Intergenerational communication, Danmaku technology, Text sentiment analysis

## Abstract

This study examines parasocial interactions (PSI) between Senior Digital Influencers (SDIs) and young audiences on the Chinese danmaku platform Bilibili. Through sentiment and content analysis of 20,728 danmaku comments, we identify key characteristics of these cross-generational PSIs. The interactions are nonlinear, bidirectional, and dominated by positive sentiments such as praise and respect. The behavioral dimension is primarily characterized by expressions of presence and companionship. The analysis reveals that danmaku technology, through its pseudo-synchronicity and anonymity, facilitates collective emotional expression and co-creates a shared viewing experience. This supports a shift from traditional one-sided PSI toward more reciprocal, co-creative engagement. The findings extend PSI theory into intergenerational digital communication. They demonstrate how platform affordances can structure positive, non-age-centric social spaces online.

## Introduction

The world is confronting a critical challenge of population aging. According to the UN DESA’s “World Development Report 2023,” the population aged 65 and above will more than double by mid-century. Protecting the well-being of older adults is therefore essential for sustainable development (United Nations, 2023).

China has the world’s largest population of older adults and is undergoing rapid demographic aging (Ministry of Civil Affairs of the People’s Republic of China, 2025). This makes the country an important context for examining issues related to aging. In the digital era, however, many older adults experience exclusion from digital life due to limited digital literacy, which can intensify intergenerational disconnection. The majority of non-Internet users in China are older adults aged over 60, a group that accounted for 62.0% of all non-Internet users (China Internet Network Information Center, 2024).

The comprehensive digitalization of daily life in China is now compelling older adults to go online, both actively and passively, to access essential social resources. This trend represents a significant wave of new senior users joining the internet, rather than merely the aging of existing users. By June 2024, China had nearly 1.1 billion internet users, with the internet penetration rate reaching 78.0%. Critically, older adults are the main driver of this growth: the 50-59 and 60+ age groups account for 15.2% and 20.8% of new users, respectively. Their adoption is driven by practical needs to simplify daily life, including the use of mobile payments, online healthcare, entertainment, social connectivity, and transport services. The high adoption rate of these services is evident: for instance, 75.4% of internet users aged 60 and above now use mobile payments (China Internet Network Information Center, 2024).

Contrary to the common assumption that older adults are disconnected from technology, a growing number are actively using social media to share their daily lives, becoming influential senior digital influencers (SDIs)—defined here as content creators aged 60 and above who share aspects of later life and have gained measurable online influence. This trend is clearly observed in China, where several SDIs have gained notable popularity on Bilibili, a Chinese video site known for its “danmaku" (bullet-screen comments) feature that enables viewers to post real-time thoughts that scroll directly over the video, thus creating a unique shared viewing experience (Bilibili Inc., 2024). The platform’s average user is around 26 years old (Nanjing Marketing Group, 2025). Those SDIs developed large-scale and positively engaged interactions with younger audience on Bilibili. This observable reality challenges existing explanations based on social identity theory, which posit that online interaction is typically characterized by in-group favoritism and out-group discrimination (Hogg, 2016; Tajfel, 1970; Turner et al., 1987). According to these views, older adults’ social circles remain largely age-homogeneous, making intergenerational connection difficult. Some research further suggests that digital environments may reproduce or even strengthen these age-based group divisions (Postmes et al., 1998).

The interactions between SDIs and younger audiences on Bilibili, however, provide a compelling counterexample. This leads to our central research question: Why are SDIs able to attract large numbers of younger audiences and sustain friendly interactions with them on danmaku platforms?

To examine this tension between theory and observation, we conducted sentiment analysis on 20,728 danmaku comments collected from SDIs’ videos on Bilibili. Our analysis examined these comments across three distinct dimensions: cognitive interactions, emotional interactions, and behavioral interactions. Through examination of these comments, we find that the danmaku medium facilitates a distinctive form of parasocial interaction that bridges generational divides through three primary mechanisms:

First, danmaku transforms solitary viewing into a collective experience. By posting and reading real-time comments, younger users create a shared, pseudo-synchronous viewing context that fosters a sense of co-presence with both the older adult creator and the viewing community. Second, danmaku comments frequently contain encouraging, empathetic, and affirming language. This direct and immediate feedback from younger viewers to older creators builds a visible, cross-generational emotional support network. Finally, SDIs actively engage with youth subcultural topics and adopt contemporary online slang. This cultural bridging strategy shifts focus away from age-based labels, grounding interactions in shared interests and values rather than age-group categories. In summary, this study extends parasocial interaction theory into the context of intergroup communication. Practically, it provides insights from the Chinese context on how digital platforms can support intergenerational integration and contribute to active aging societies.

## Literature Review

### Social Identity Theory and Group Distinction

Previous theories often posit that older adults tend to interact more within their own age group rather than with younger people. Social identity theory (Tajfel, 1970) provides a foundation for this phenomenon. The theory suggests that mere intergroup categorization is sufficient to trigger ingroup favoritism and outgroup discrimination (Billig & Tajfel, 1973). Social norms of “group belonging” further guide such discriminatory behaviors. Tajfel (1972) defined social identity as an individual’s knowledge of belonging to certain social groups, combined with the emotional and value significance of that membership.

Social categorization means people are understood not just as individuals, but as members of social categories, including age groups (Trepte & Loy, 2017). Research since the 1980s shows that human groups engage in self-categorization. This process forms a relatively stable core for the ingroup, while making the differences between the ingroup and outgroup more specific (Hogg, 2016; Turner et al., 1987).

The distinctions between older and younger adult groups are well-documented (Celejewski & Dion, 1998; Chasteen et al., 2002). Prejudice against older adults often involves perceptions of incompetence, disrespect, and pity (Fiske et al., 2018). This prejudice can drive older adults to behave in ways that align with their age group’s stereotypes, potentially to mitigate the negative effects of discrimination (Chasteen, 2005).

However, some evidence offers a more positive perspective on breaking group distinctions. Matheson et al. (2000) found that older adults held multiple stereotypes about younger adults, with positive stereotypes outweighing negative ones. Chasteen (2005) also observed that while both young and old adults showed ingroup favoritism and outgroup bias, older adults exhibited less bias, potentially due to their greater familiarity with the outgroup. Despite these positive nuances, the fundamental persistence of age-based social categorization is a common acknowledgement across these studies.

The inferences of social identity theory also find support in the context of digital communication. Postmes et al. (1998) found that digital technology significantly implicates social processes. It can accentuate the influence of pre-existing social boundaries from the real world and potentially erect new ones.

### Parasocial Interaction and Identification

Parasocial interaction (PSI) refers to the one-sided, illusory sense of connection that media audiences develop toward media personae (Horton & Wohl, 1956). A key driver behind this process is identification, where audiences come to see themselves in relation to a media figure (McQuail & Blumler, 1972).

Kelman (1958, 1961) conceptualized identification as a form of social influence. Through it, individuals internalize the attitudes, values, and beliefs of another to maintain a desired relationship with that figure, often adopting their behaviors as well (Kelman, 1961). This “self-defining” relationship aligns closely with the notion of a parasocial relationship (Brown, 2015). Media users actively seek out personae with whom they identify, and in doing so, may adopt those figures’ worldview and conduct (Burke, 1969).

Identification can also be a long-term outcome of PSI. Cohen (2001) argues that repeated internalization of a character’s identity and perspective can lead to enduring psychological effects. He further explains that identification involves sharing a character’s viewpoint, feeling with them, and imagining oneself as part of their narrative world (Cohen, 2001).

The relationship between identification and PSI suggests two potential pathways for inter-age group dynamics online. First, individuals may extend their existing age-based identifications into the digital space, forming PSIs only with personae similar to themselves, thereby reinforcing existing social categorizations. Alternatively, people may develop PSIs with media figures from different age groups, leading to identifications that transcend their original social identities. This process can foster attitudes and behaviors that bridge traditional group boundaries.

In the context of Bilibili, the PSIs formed between younger audiences and senior digital influencers (SDIs) illustrate a promising case of cross-age understanding. By engaging with content created by SDIs, younger audience can develop empathetic identification with older adults. Such connections offer a practical basis for reducing group-based discrimination and open new possibilities for intergenerational dialogue.

### Parasocial Interaction in the Digital Age and Danmaku Technology

Traditional parasocial interaction (PSI) theory emphasizes the one-sided influence of a media persona on the audience. In contrast, the PSI between senior digital influencers (SDIs) and younger audiences on danmaku platforms demonstrates how digital technology can facilitate more reciprocal engagement. This suggests that audiences can also exert significant and real-time influence on media personae.

Originally, PSI was considered inherently unilateral due to the one-way nature of broadcast media. In this model, audiences developed strong feelings or attachments toward personae, who could not directly respond. Thus, the relationship was understood as imaginary and unidirectional (Horton & Wohl, 1956). Both positive and negative effects of such one-sided PSI have been observed, though these effects were confined to the audience. While media personae might eventually be affected by collective audience feedback, such influence was typically asynchronous and not transmitted in real time. Early research, such as McQuail and Blumler’s (1972) study on soap operas, highlighted how audiences empathized with on-screen characters, yet this engagement remained psychologically distant.

The emergence of interactive social media has fundamentally reshaped this dynamic. Platforms that enable comments, live chats, and other feedback mechanisms have transformed PSI from a static reception experience into a more dynamic and seemingly reciprocal process (Giles, 2002). This shift challenges the traditional view of PSI as a purely one-dimensional phenomenon and underscores that audience involvement is no longer just a solitary psychological act (Brown, 2015). In this new environment, various forms of mediated relationships have emerged. They range from brief interactions to sustained parasocial attachments (Stever & Lawson, 2013). These are particularly evident in contexts like live streaming, where interactions are often gamified and commodified (Li, 2015; Wang, 2019). Reflecting these developments, Lou (2022) proposed the concept of “trans-parasocial relations,” which are collectively reciprocal, synchronously interactive, and co-created. Although this concept has been primarily applied to consumption contexts like live-streaming commerce rather than well-being, it nonetheless marks a significant departure from the traditional one-sided model by emphasizing dynamic collaboration between audiences and influencers.

Among various digital technologies, danmaku offers a distinctive medium that exemplifies the features of trans-parasocial interaction. While other comment systems also offer co-viewing experiences, danmaku is unique in several ways. First, it supports collective reciprocity. Danmaku comments are fully anonymous, reducing constraints associated with self-identity and potentially encouraging more honest and unrestricted self-expression (Chen et al., 2017). This fosters more authentic dialogue, free from the need to maintain a fixed social identity. Second, danmaku is synchronously interactive. It provides a “pseudo-synchronous” co-viewing experience (Johnson, 2013), allowing viewers to see comments left by all previous viewers at specific moments in the video. Third, it is co-creative. Comments are visually embedded and overlaid on the video, transforming the original content into a new collaborative work that merges informational, and entertainment value contributed by both creators and viewers.

As one of the largest danmaku video platforms in China (Bilibili Inc., 2024), Bilibili offers an ideal context to observe trans-parasocial interaction in practice. The interactions between SDIs and younger audiences on Bilibili are likely to lead to more enduring, profound, and authentic changes in cognition, emotion, and behavior. This is particularly true in fostering mutual understanding and shared meaning-making across generations.

### Parasocial Interaction and Active Aging

Parasocial interaction (PSI) has long been recognized for its influence on the well-being of older adults. Research indicates that those lacking fulfilling real-life relationships are more inclined to develop PSI, which can significantly shape their emotional and social lives (Horton & Wohl, 1956; Tsao, 1996). Such mediated relationships often provide companionship and help restore a sense of intimacy that may have been diminished by life transitions such as retirement or loss of partners (Lim & Kim, 2017; Stever, 2008, 2011). However, PSI does not uniformly benefit older adults. For those experiencing high anxiety within low-quality real-life relationships, parasocial engagement may even exacerbate depressive symptoms (Bernhold & Metzger, 2020).

A critical and emerging question is how the transformation of PSI may reshape the aging experience. Enabled by digital technology, this transformation moves from a one-sided broadcast dynamic to an interactive, bidirectional process. This question is particularly relevant given the increasing role of video media in the daily lives of older adults, many of whom now treat it as a substitute for declining offline social interaction. A recent report on Internet usage among older adults in China revealed that 43% of elderly users spend more than four hours per day watching short videos (The Beijing News, 2024).

The active aging framework emphasizes sustained social participation, psychological well-being, and personal autonomy in later life. From this perspective, the recent rise of senior digital influencers (SDIs) represents a significant shift. Rather than being passive media consumers, these older adults are becoming active participants in the digital public sphere. By creating content and building communities, they engage in meaningful social interaction, maintain cognitive and emotional activity, and exercise personal agency. These are all core components of active aging. This form of participation allows them to counteract ageist stereotypes and contribute to society in new, digitally mediated ways, thereby supporting both social integration and psychological well-being.

Therefore, examining PSI in the context of SDIs not only updates traditional communication theories but also offers a novel lens through which to study active aging in the digital era. This lens highlights empowerment, community engagement, and the positive use of technology in later life.

Based on the literature reviewed above, our study proposes the following research questions:

**RQ1**: What are the defining characteristics of parasocial interaction (PSI) between senior digital influencers (SDIs) and younger audiences in the digital media context?

**RQ2**: How do the technological and communicative features of danmaku platforms shape these PSI characteristics?

Based on the results of RQ1 and RQ2, this study also want to discuss how these emerging forms of PSI, as reflected in danmaku interaction, influence older adults’ active aging.

## Methodology

This study analyzed the sentiment analysis of the danmaku of Bilibili videos posted by SDI. The research design is as follows.

### Introduction to the Scales

The measurement of parasocial interaction has been developed through several key scales. Horton and Wohl (1956) divided parasocial interaction into cognitive interaction, emotional interaction and behavioral interaction. Building on Levy’s (Levy & Windahl, 1984) seven indicators, Rubin et al. (1985) developed the Parasocial Interaction (PSI) scale in 1985, which became a widely used benchmark. Later, Auter and Palmgreen (2000) formulated the 22-item Audience-Persona Interaction (API) scale in 2000 to measure long-term engagement. In 2008, Schramm and Hartmann (2008) proposed a 12-item instrument assessing cognitive, behavioral, and affective dimensions. To specifically measure parasocial relationships, Tukachinsky (2010) introduced a 24-item scale in 2010, identifying quasi-friendship and quasi-romance as key components. A further development was the Experience of Parasocial Interaction (EPSI) scale by Hartmann and Goldhoorn (2011) in 2011, who applied a social psychological perspective. As validated by Dibble et al. (2016), the EPSI scale captures the core experience of parasocial interaction more directly than the earlier PSI scale, which often reflects general liking.

Due to space limitations, this section only introduces the most representative scales (Table 1).

**Table 1:** PSI scales introduction

Scale Type	Proponent(s)	Description
PSI Scale	Schramm and Hartmann (2008)	Cognitive
		*∙* Attention allocation
		*∙* Comprehension of persona’s action and situation
		*∙* Activation of prior media and life experience
		*∙* Evaluations of persona and persona’s actions
		*∙* Anticipatory observation
		*∙* Construction of relations between persona and self
		Affective
		*∙* Sympathy/antipathy
		*∙* Empathy/counter empathy
		*∙* Emotion contagion
		Behavioral
		*∙* Nonverbal behavior (e.g., mimics, gestures)
		*∙* (para-)verbal behavior
		*∙* Behavioral intentions
EPSI Scale	Hartmann and Goldhoorn (2011)	While watching the clip, I had the feeling that the persona:
		1. was aware of me
		2. knew I was there
		3. knew I was aware of him/her
		4. knew I paid attention to him/her
		5. knew that I reached to him/her
		6. reacted to what I said or did

Subsequent scholars have continued to adopt the classical division logic of PSI established by the aforementioned scholars. Ge and Fang (2010) and Ge (2017) and others introduced the scale to China, adapted it for local contexts, and developed Likert and Guttman scales for parasocial interaction and relationships. Ma et al. (2020) adopted Ge and Fang’s Chinese parasocial relationship scale to explore the subjective well-being of live-streaming users. Using random sampling and a Likert scale, Lim et al. (2020) found that audience identification with and emotional investment in streamers fosters parasocial relationships, thereby enhancing user stickiness. Focusing on local hosts, Ma (2007) argued that cognitive interaction between media figures and audiences involves information acquisition and perspective comparison centered on content; attitudinal interaction manifests in mutual emotional exchange; and behavioral interaction refers to “the public’s internalization of hosts’ behaviors.” Rubin and Perse (1987) analyzed audience parasocial interaction with TV drama characters across three dimensions: cognition, behavior, and emotion. This study retains the established three-dimensional framework of parasocial interaction while introducing further connotative innovations to align with the latest online contexts.

### Data Collection

**Table 2:** Profile and video data of SDIs

SDI	Video Category	Video Content (ID)	Views	Likes
			(in 10k)	(in 10k)
YWYY	Harmonica Performance	Playing Popular Songs on Harmonica (YWYY1)	38.2	4.1
(Male, Unknown)		Playing Popular Songs on Harmonica (YWYY2)	31.0	3.4
		Harmonica Skills Tutorial (YWYY3)	28.8	1.0
SSNNT	Fashion & Style	Elegant Outfit Sharing (SSNNT1)	53.2	2.8
(Female, 67)		JK Uniform Outfit Sharing (SSNNT2)	51.3	4.3
		Fashion Photography (SSNNT3)	43.5	4.5
DJY	Classical Poetry Explanation	Poetry Analysis (DJY1)	572.5	37.2
(Male, 69)		Travel & Food Vlog (DJY2)	224.8	11.0
		Sharing Emotional Memories (DJY3)	189.6	21.9
YLT	Video Gaming	Buying a Game Console (YLT1)	191.2	5.6
(Male, 89)		Receiving the Game Console (YLT2)	133.9	6.8
		Gameplay Recording (YLT3.1)	116.4	4.6
		Sharing Game Completion Experience (YLT3.2)	103.1	3.0
YYTLY	Comedy & Everyday Vlogs	Recording the SDI’s Passing (YYTLY1)	172.2	15.4
(Male, 87)		Pranking the SDI (YYTLY2)	91.8	4.5
		Pranking the SDI (YYTLY3)	91.6	4.2
MCBL	Life Experience Sharing	Starting as an SDI at Age 90 (MCBL1)	558.4	63.5
(Female, 94)		Food Making Sharing (MCBL2)	155.8	12.7
		Sharing Memories of the War of Resistance (MCBL3)	91.0	17.9

To further refine the dataset, special event videos, collaboration videos, and promotional videos produced by these SDIs were excluded. Their remaining works were then categorized by theme and ranked according to view counts. For each SDI, three videos with distinct themes and the highest view counts were selected, yielding a total of 18 videos for analysis (Table 2).

We employed a “keyframe” technique to analyze the video language, expressions, and movements of the SDIs frame by frame. The relevant data will be referenced in the Results section to support the demonstration of real-time interaction dynamics between the SDIs and their audiences via danmaku comments. However, due to space constraints, these visual materials are not presented here.

In addition, all danmakus associated with these videos were collected using Python-based web-scraping tools. After removing duplicate entries, the dataset comprised a total of 20,728 unique danmaku texts, as shown in Table 3.

**Table 3:** Sample size

No.	SDI	Danmaku Count
1	DJY	5186
2	YLT	3726
3	YWYY	2419
4	SSNNT	2997
5	YYTLY	1375
6	MCBL	5025
7	Total	20728

#### Sample Characteristics

To address the concern that the age of commenters cannot be inferred solely from platform-level demographics, we conducted a supplementary content analysis directly on the danmaku comments. We adopted a stratified sampling strategy, selecting 30 comments from each of the six SDIs, yielding a total of 180 comments. After removing non semantic comments (e.g., “hahaha”, *n*=5), 175 valid comments remained. We coded each comment based on four linguistically identifiable markers of youth (see Table 4). Two coders independently rated the comments, and inter coder agreement exceeded 80%. Among the 175 valid comments, 119 (68.0%) were identified as definitively from younger audiences based on at least one of the four criteria. For the remaining 56 comments (32.0%), no definitive age marker was present. However, our coding of the comments did not identify any explicit indicators associated with older adulthood. Specifically, we coded for self-references related to age (e.g., being retired or identifying as an older adult), family-role references associated with older age (e.g., grandparental roles), age-related scenarios, and other age-specific linguistic cues.

**Table 4:** Criteria for identifying younger audiences based on danmaku content

Criterion	Example	Explanation
Internet slang	Failed to freeload(YWYY)	A Bilibili-specific expression indicating that the content was so excellent that the viewer felt compelled to support the creator.
Use of honorifics	Grandpa, rest in peace (YYTLY)	Addressing the SDI with a respectful term, implying deference and familial affection typical of younger generations.
Youth-relevant scenarios	Feels like I am being urged to marry (DJY)	A common experience among younger adults (e.g., parental pressure to marry), which reasonably suggests a younger commenter.
Youth-typical tone	My own grandpa said he can do it (SSNNT)	The commenter refers to their own grandfather, implying a younger generational position relative to the SDI.

### Classification Criteria for Parasocial Interaction: Cognitive-Behavioral-Emotional

Building upon the theoretical and applied contributions of scholars such as Horton and Wohl (1956), Schramm and Hartmann (2008) to Parasocial Interaction (PSI) theory (Ge & Fang, 2010; Lim et al., 2020; Rubin & Perse, 1987), this study adopts the cognitive-behavioral-affective interaction framework. By integrating the context of new media and the distinctive features of Chinese danmaku, it provides a constructive extension and refinement to this three-dimensional PSI model. The specific dimensions are as follows: **Cognitive Interaction** into *perception and evaluation, memory recall, social comparison, role identification, recognition and expectation*, and *information exchange and topic discussion*. **Behavioral interaction** is categorized into *presence and companionship, imitation and behavioral motivation*, and *following and supportive actions*. **Emotional interaction** is categorized into *empathy*, *emotional contagion*, and *collective mourning* (Table 5).

**Table 5:** Parasocial interaction cognitive-behavioral-emotional categories

Cognitive Interaction	
Perceptual Evaluation	The audience’s intuitive feelings and evaluations of senior digital influencers (SDIs), and their understanding of SDI’s language behavior.
Memory Evocation	The audience recalls or associates things related to the video content while watching the SDI’s work.
Social Comparison	The audience compares their own or their family’s abilities, status, or situation with that of the SDI.
Role Identification	The audience projects their own social identity onto the SDI, their close relatives, or other characters in the video.
Recognition and Expectation	The audience agrees with the SDI’s opinions and performances and hopes for continued video updates.
Information Exchange and Topic Discussion	This includes both the new knowledge or skills the audience gains from the SDIs, and the additional information or background knowledge the audience provides to help the SDI and other viewers better understand the work; topic discussions refer to the audience’s opinions and extended discussions on the topics related to the SDI.
Behavioral Interaction	
Presence and Companionship	The audience experiences an immersive feeling in the SDI’s videos, as if the SDI is accompanying them, engaging in daily conversations with the SDI.
Imitation and Behavioral Motivation	The audience is influenced by the SDI, imitating their words (including witty phrases, concise words, emphasis, catchphrases, etc.), or intends to change and reshape their own real-life behavior by referencing the SDI’s actions and habits.
Following and Supportive Actions	Following refers to: online, the audience follows the same SDI across platforms and programs; in real life, the audience wishes to meet or encounter the SDI; support refers to: the audience express support for the SDI through words or actions such as liking, sharing, or bookmarking.
Emotional Interaction	
Empathy	The audience empathizes with the SDI’s inner world, contemplating their thoughts, understanding their situation, and conveying this understanding to the SDI.
Emotional Contagion	The audience is emotionally influenced by the SDI, empathizes, and expresses corresponding emotions.
Collective Mourning	Due to the SDI’s passing, the audience spontaneously organizes collective expressions of mourning.

For example, in video DJY1, the danmaku comment “Hearing the teacher say this line made me cry” belongs to Emotional Interaction - Emotional Contagion. In video MCBL1, the danmaku comment “Grandma is more stylish than I am” falls under Cognitive Interaction - Social Comparison.

We employed a chi-square test to examine the differences in the distribution of danmaku comments across the three interaction types (cognitive, behavioral, and emotional) among different SDIs. The result ($\chi ^2 = 6279.05$, *p < 0.001*) indicates a statistically significant difference in the distribution of interaction-type danmaku across different SDIs.

### Sentiment Analysis

The sentiment lexicon used in this study was primarily based on the Sentiment Ontology developed by Xu’s research team at Dalian University of Technology (Xu et al., 2008). This ontology classifies sentiments into seven major categories: Joy (happiness, security), Good (praise, trust, respect, affection, touched, well-wishing), Anger (anger), Sadness (sadness, disappointment, guilt, yearning), Fear (panic, fear), Disgust (shame, annoyance, criticism, disgust, jealousy, suspicion), and Surprise (surprise).

To better adapt the lexicon to the context of danmaku comments, this study introduced a new dimension, “Teasing,” under the Joy category. An additional 785 terms were incorporated into the lexicon, based on a dataset of danmaku comments that revealed high-frequency emerging internet slang not covered in the original lexicon. For example, xswl, an abbreviation for laughing to death, is categorized under *Happiness*.

The classification of specific terms considered both their frequency and contextual use. For instance, the term wuwu (was categorized as “touched,” as seen in danmaku comments like *wuwu, I’m so touched*. Similarly, *leibeng* (meaning burst into tears), was classified under *Sadness*, corresponding to comments such as *This is so painful, I want to cry, burst into tears*.

Additionally, the original sentiment lexicon includes part-of-speech tagging for various types of expressions, such as nouns, verbs, adjectives, idioms, colloquial phrases, and prepositional phrases with emotional connotations. This study preserved as much of the original data as possible and manually annotated the newly added terms with corresponding parts of speech to ensure consistency and accuracy (Table 6).

**Table 6:** Example of newly added sentiment words

No.	Sentiment word	Word type	Sentiment type
1	xswl	Internet Neologism	Happiness
2	doge	Internet Neologism	Teasing
3	brilliant man (niu-ren)	Internet Neologism	Praise
4	as same as me (an-ye-yi-yang)	Phrase	Trust
5	the older gentelman (lao-ren-jia)	Noun	Respect
6	like (dian-zan)	Verb	Affection
7	cry (wu-wu)	Verb	Touched
8	jealous (suan-le)	Internet Neologism	Admire
9	longlive (chang-ming-bai-sui)	Idiom	Well-wishing
10	surprised (jing-dai-le)	Internet Neologism	Surprise

This study uses an exact matching word segmentation method, utilizing the Jieba tokenizer in Python. In exact matching, the mutual exclusivity between words is first considered, and the data is organized accordingly. For example, both *xiaohaha* (laughing loudly) and *ha-ha* express a joyful sentiment, but in exact matching, *ha-ha* is retained to encompass more synonyms.

#### Reliability Test of the Three-Dimensional Scale

We randomly selected 50 entries from each of the six SDI datasets, yielding a total of 300 entries (1.4% of the total sample). Intercoder reliability for the three core dimensions of parasocial interaction was assessed using Cohen’s Kappa coefficient. The evaluation examples and results are presented in Table 7.

**Table 7:** Intercoder reliability (Cohen’s Kappa) for cognitive, behavioral, and emotional interactions

Category	Cohen’s *κ*	95% CI	Agreement %
Cognitive Interaction	0.87	[.82, .91]	93%
Behavioral Interaction	0.82	[.77, .86]	90%
Emotional Interaction	0.89	[.85, .92]	94%
Overall (Average)	0.86	[.83, .89]	92%

The results indicate that all coefficients significantly exceed the commonly accepted threshold of 0.8, demonstrating that the coding scheme exhibits good consistency and reliability.

####  Reliability Test of Sentiment Lexicon

A random sample of 100 entries was selected from the newly added pool of 785 sentiment words, accounting for 12.7% of the total lexicon. The primary and secondary researchers independently completed the coding for this subset. The inter-coder reliability was then calculated using the Spearman-Brown prediction formula, a classic psychometric method for estimating the reliability of a measurement system based on inter-coder agreement. This approach is well-established in the methodological literature of psychology for assessing coding consistency. Specifically, we adopted the procedure outlined in Dong’s (2004) authoritative work, *Psychological and Educational Research Methods*, which presents this formula as a standard for reliability estimation in psychosocial content analysis. This method is preferred because it moves beyond simple percentage agreement by providing a standardized estimate of the coding scheme’s overall reliability.The specific coding examples and the reliability results are presented in Table 8:

**Table 8:** Reliability test results for the sentiment lexicon

Sample	Sentiment Word	Primary Coder’s Label	Secondary Coder’s Agreement
Sample 1	you (nin)	Respect	Yes
Sample 2	sigh (ai)	Sadness	No
Sample 3	hard work (xin-ku)	Praise	Yes
Sample 4	veteran player (lao-wan-jia)	Praise	Yes
Sample 5	sober (qing-xing)	Affection	Yes
... ... ...			
Sample 98	HH (laughter)	Happiness	Yes
Sample 99	core player (he-xin-wan-jia)	Good	Yes
Sample 100	assist (zhu-gong)	Good	Yes
Overall Reliability:			98.9%

The results show a reliability score of 98.9% for the sentiment lexicon, thus confirming the credibility and consistency of the primary researcher’s categorization.

#### Sentiment Computation

##### Sentiment Type

The sentiment type reflects the primary emotion conveyed in a given danmaku comment. While a comment may contain multiple types of emotions, only one primary sentiment is assigned.

##### Calculation Method for Sentiment Type

The sentiment type of a comment is determined by calculating the word frequency of sentiment words within the comment using computational analysis. The sentiment category corresponding to the highest word frequency is assigned as the primary sentiment type. The calculation formula is as follows:

1 $$\begin{aligned} \text {Sentiment}_1 = \max (N_i) \end{aligned}$$

where $Sentiment_{1}$ represents the primary sentiment type of an individual comment, and $N_{i}$ represents the word frequency for a specific sentiment category. The value of *i* ranges from 1 to 22, corresponding to 22 sentiment categories, as detailed in the Table 9.

**Table 9:** Sentiment category value range

i value	1	2	3	4	5	6
Sent type	Happiness	Security	Teasing	Praise	Trust	Respect
i value	7	8	9	10	11	12
Sent type	Affection	Admire	Well-wishing	Anger	Sadness	Disappointment
i value	13	14	15	16	17	18
Sent type	Guilt	Missing	Fear	Shame	Annoyance	Criticism
i value	19	20	21	22		
Sent type	Disgust	Jealousy	Suspicion	Surprise		

##### Sentiment Polarity

Sentiment polarity reflects whether the sentiment conveyed in a single danmaku comment is positive or negative, as well as the intensity of the sentiment. Based on the categorization in the sentiment lexicon and the specific context of this study, emotions such as *happiness, praise, respect,* and *surprise* are classified as positive emotions, while emotions such as *sadness, criticism, annoyance*, and *fear* are categorized as negative emotions. In this study, each occurrence of a positive sentiment word within a comment is assigned a score of +1, while each occurrence of a negative sentiment word is assigned a score of -1. If a positive sentiment word and a negative sentiment word appear simultaneously in the same comment, the score is recorded as 0. We category *surprise* as a positive emotion. This decision is based on the overall sentiment representation of *surprise* in danmakus, where it predominantly reflects emotions such as delight and astonishment, rather than fear or panic, which carry negative connotations (Table 10).

$Sentiment_{2}$ represents sentiment extremity. $Q_{pos}$ represents the number of positive sentiment words in a single danmaku, with each occurrence assigned a score of +1. $Q_{neg}$ represents the number of negative sentiment words in the comment, with each occurrence assigned a score of -1. When the number of positive and negative sentiment words is equal, the score is 0.

2 $$\begin{aligned} \text {Sentiment}_2 = {\left\{ \begin{array}{ll} Q_{\text {pos}} & Q_{\text {pos}}> Q_{\text {neg}} \\ 0 & Q_{\text {pos}} = Q_{\text {neg}} \\ -1 \times Q_{\text {neg}} & Q_{\text {pos}} < Q_{\text {neg}} \end{array}\right. } \end{aligned}$$

**Table 10:** Example table of danmaku sentiment value calculation

SDI	Danmaku	Word	Type	Polarity
DJY	My father just passed away. He used to sneak into my room to turn off the light. If he saw that I was still awake, he would scold me. It was so annoying at that time. But now I can never see him sneak into my room and blame me again: “Why aren’t you asleep yet?” I feel so sad. This is probably what people mean by “only realizing the ordinary at that time.”	father: Respect passed away: Sadness scold me: Annoyance annoying: Disgust blame: Annoyance sad: Sadness only realizing the ordinary at that time: Sadness	Annoyance	*-5*

## Result

### Parasocial Interaction Dimensions Overall Map

**Fig. 1:**
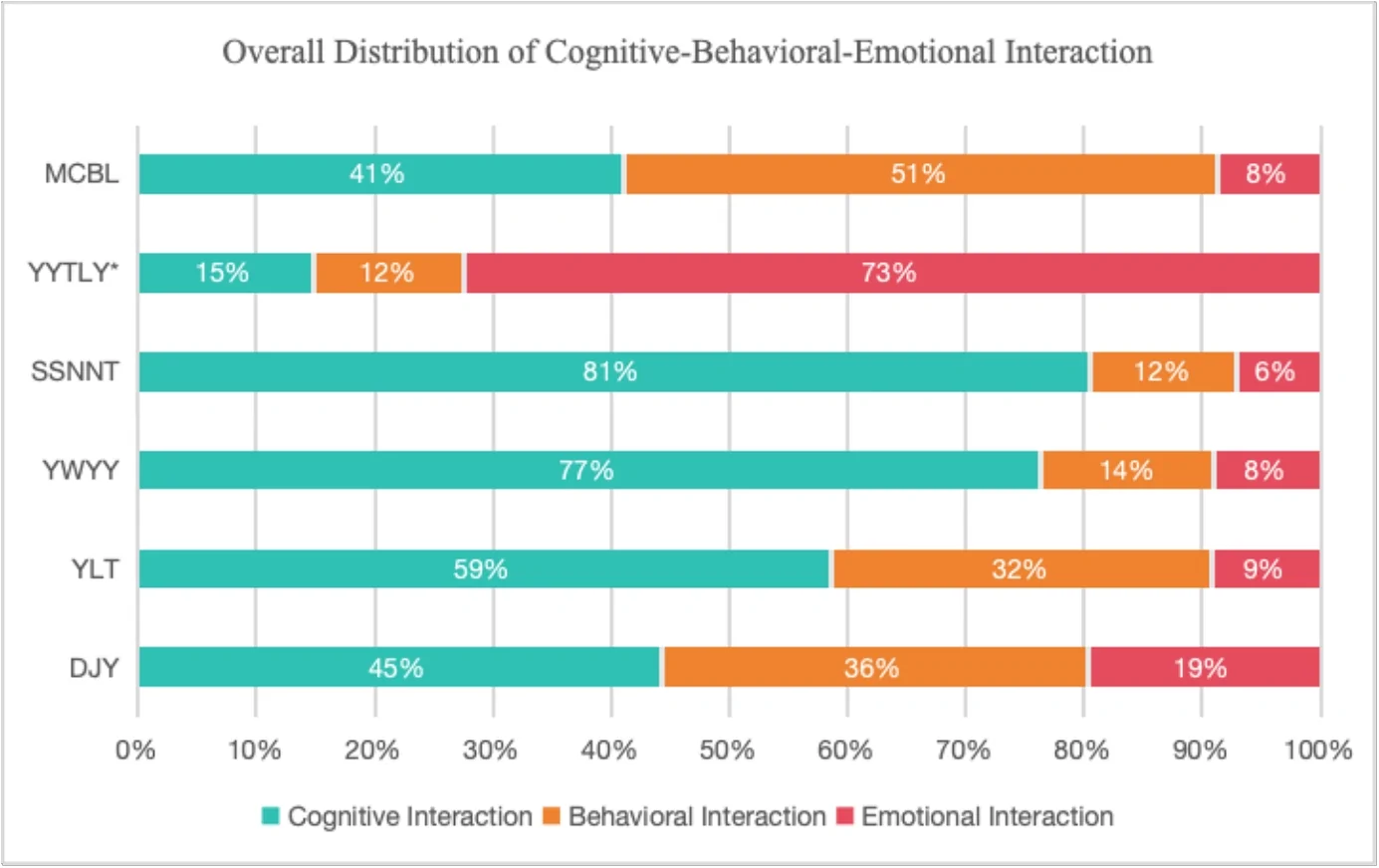
Overall distribution of cognitive-behavioral-emotional interaction

In the parasocial interactions between Senior Digital Influencers (SDIs) and their audiences, cognitive interaction was the most prevalent dimension, followed by behavioral interaction. Emotional interaction was the least common type observed. Analysis across all six SDI cases confirmed that each exhibited all three types of interaction simultaneously. A notable finding was the substantial proportion of behavioral interactions in several cases. For the SDI MCBL, behavioral interactions constituted 51% of total interactions, exceeding both cognitive (41%) and emotional (8%) dimensions. The distribution of interaction types, however, varied significantly among individual SDIs. A distinct pattern emerged for YYTLY. In danmaku comments on videos posted prior to his passing, emotional interactions were predominant at 73%, far surpassing cognitive interactions at 15%. This stands in clear contrast to the behavioral-led pattern observed for MCBL (Fig. 1).

Within the cognitive dimension, the sub-categories of perception and evaluation together constituted the majority (over 50%) of interactions. A notable thematic pattern emerged for SDIs YYTLY and DJY, who exhibited a significantly higher proportion of memory recall compared to other influencers, a finding associated with content related to loss and remembrance.

The emotional dimension comprised empathy, emotional contagion, and collective mourning. Emotional contagion was the most prevalent sub-category across SDIs, accounting for 55% to 99% of emotional interactions. Empathy represented a smaller but consistent proportion (3% to 45%), with its salience particularly noted in responses to SDI MCBL, often triggered by visible signs of aging such as trembling hands. The collective mourning sub-category appeared uniquely and dominantly in interactions involving YYTLY (73% of his emotional interactions) and DJY (71% of his emotional interactions), who shared content directly addressing themes of death and personal loss.

**Fig. 2:**
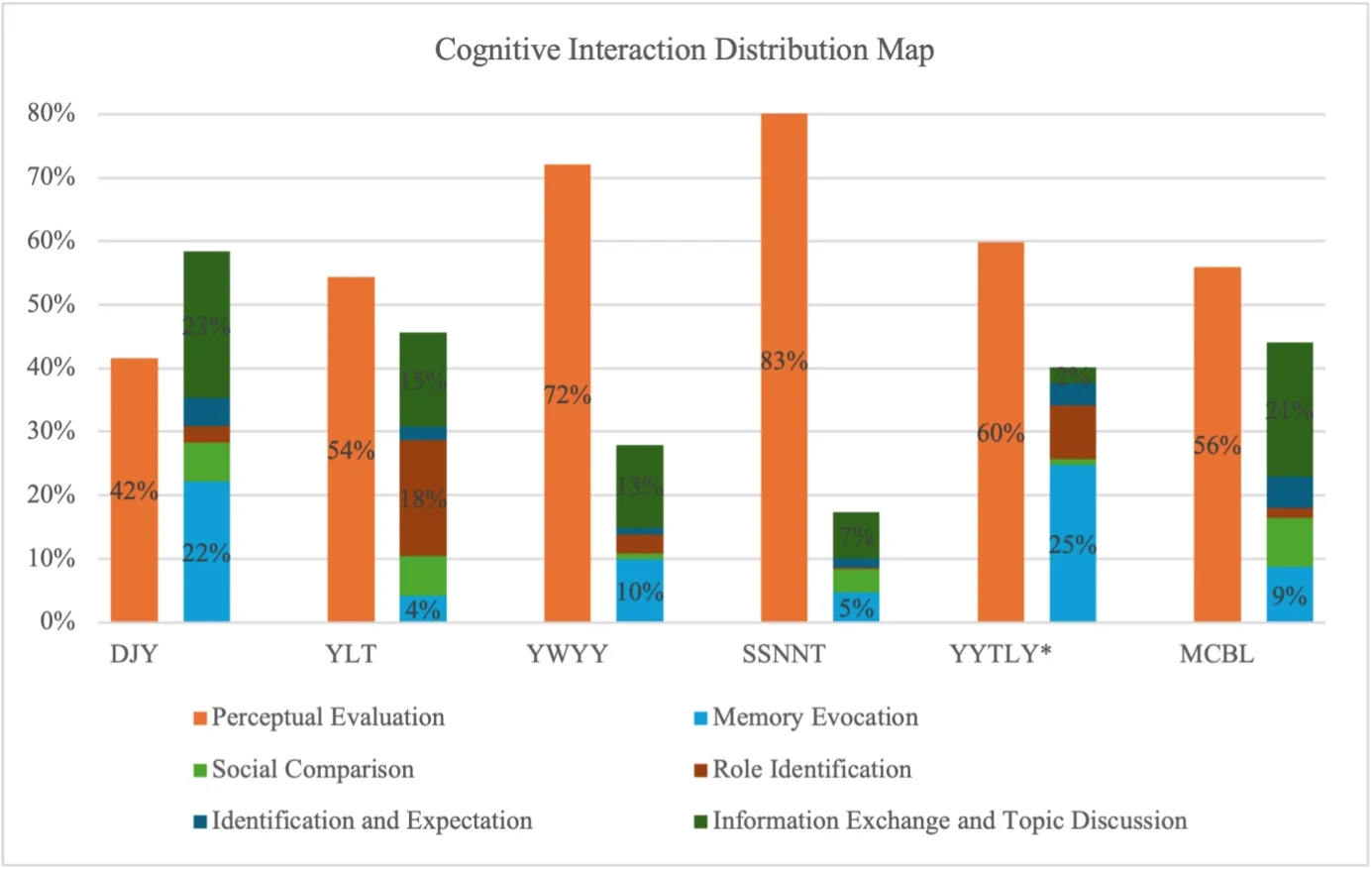
Cognitive interaction distribution map

**Fig. 3:**
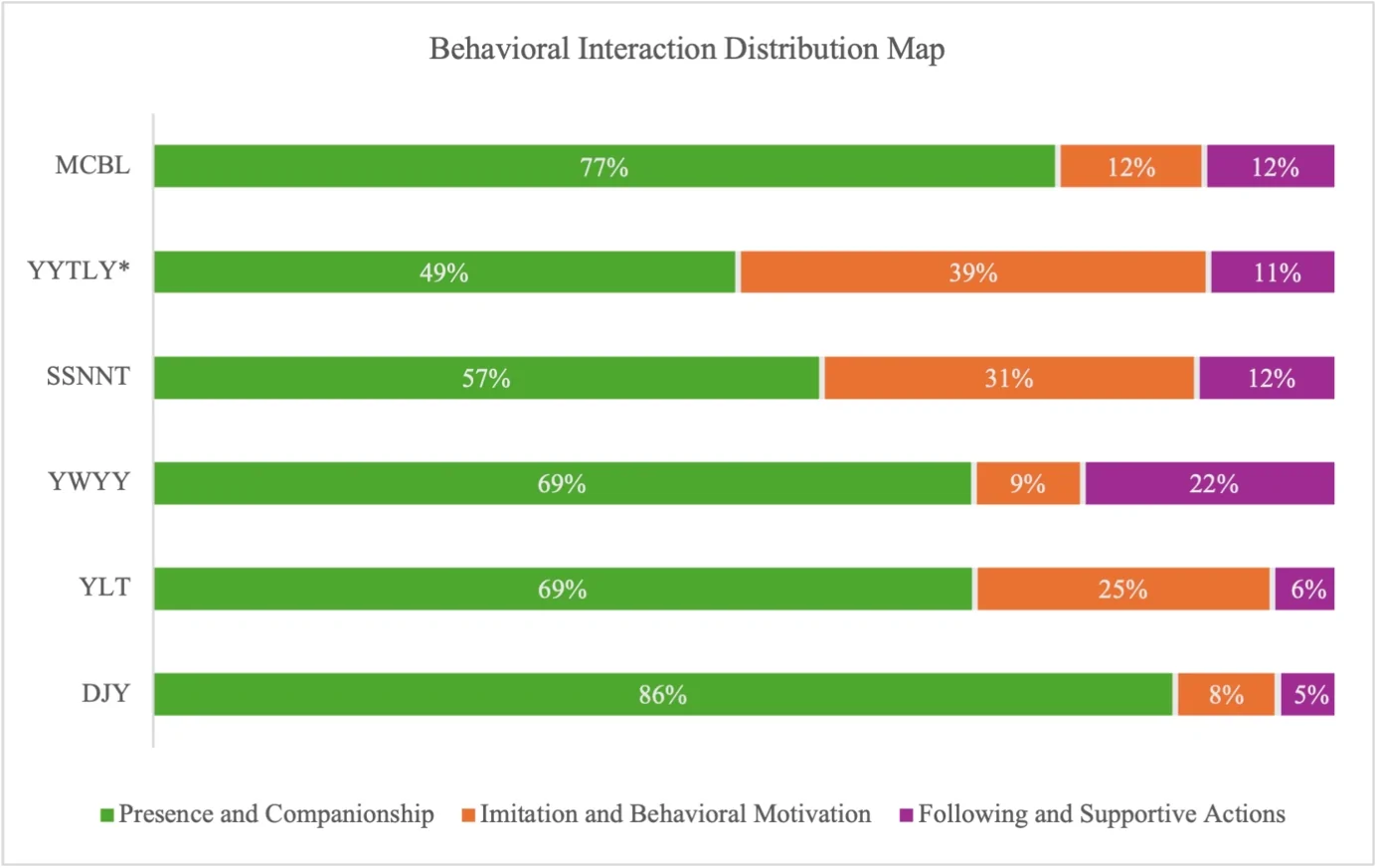
Behavioral interaction distribution map

**Fig. 4:**
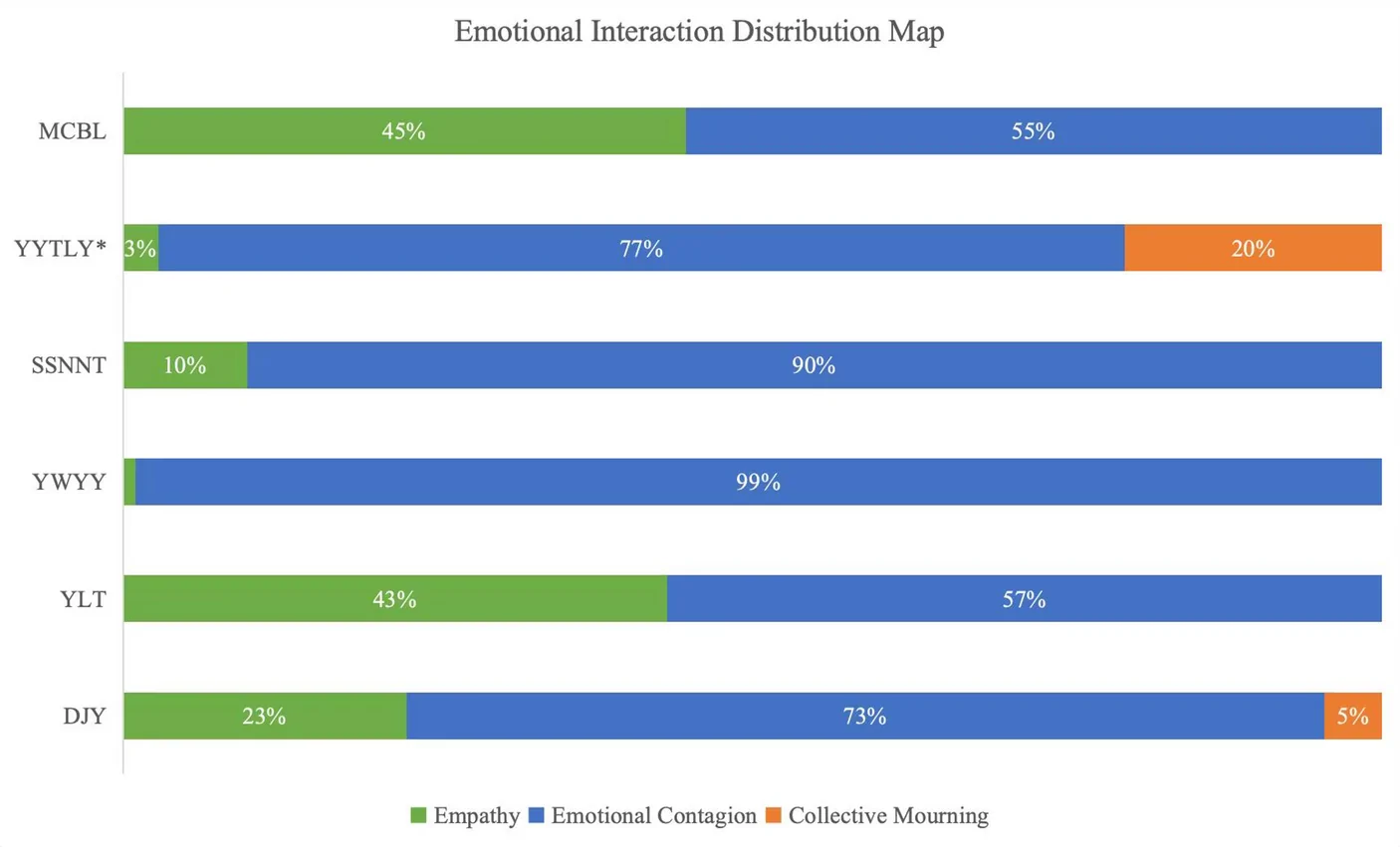
Emotional interaction distribution map

In the behavioral dimension, presence and companionship was the dominant sub-category across all SDIs. The distribution of other behavioral sub-categories (imitation and behavioral motivation; following and supportive actions) varied distinctly according to the SDI’s persona and content theme. For instance, YYTLY (entertainer, daily funny life) elicited the highest proportion of imitation and behavioral motivation, whereas DJY (scholar, poetry appreciation) triggered the most pronounced presence and companionship responses but the least imitation (Figs. 2, 3, and 4).

### Temporal Analysis

**Fig. 5:**
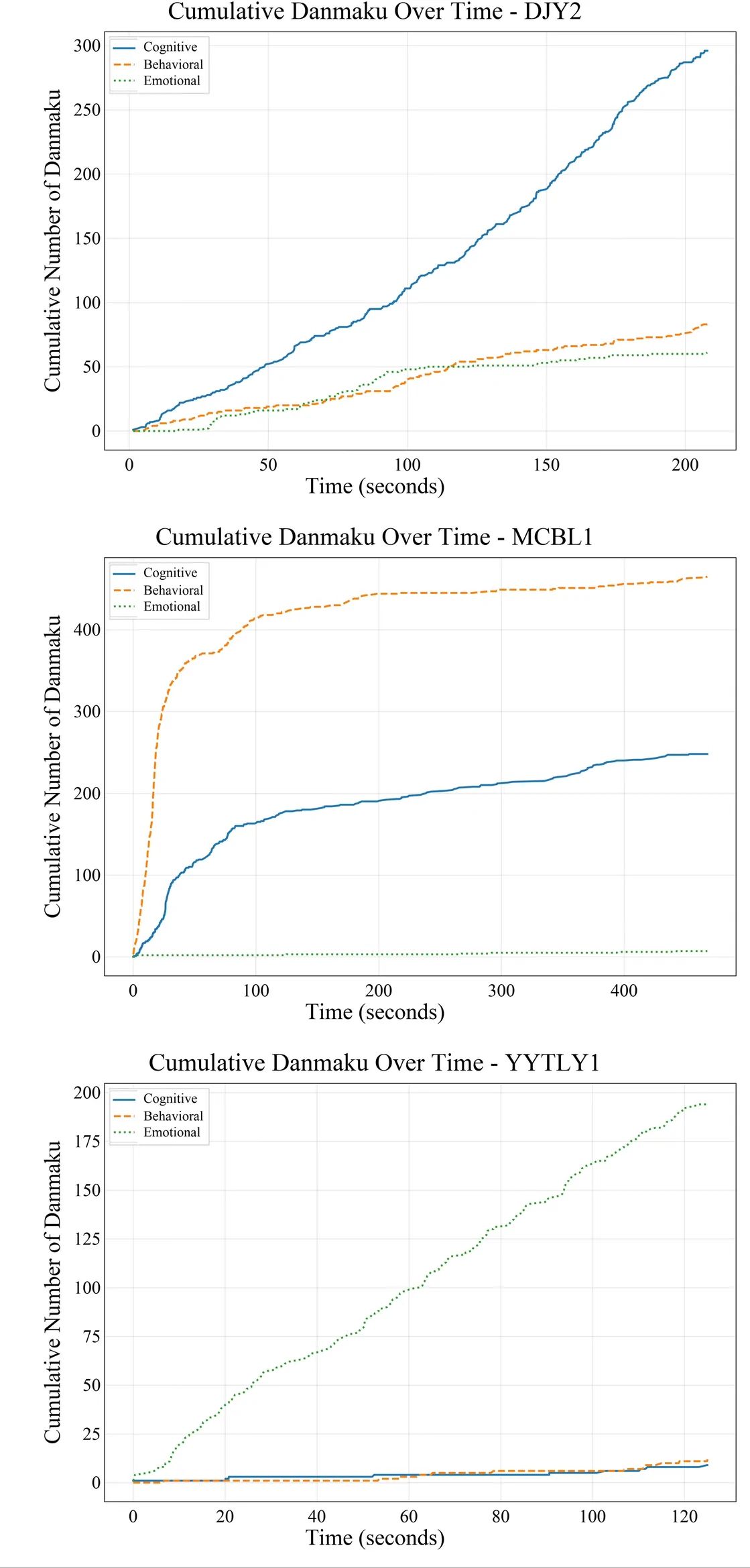
Temporal analysis of danmaku interaction types for typical videos by SDIs

The temporal analysis of danmaku comments across representative videos (see Fig. 5) reveals that cognitive, behavioral, and emotional interactions are closely tied to the specific content of the video narrative. Their developmental trajectories do not follow the traditional linear “cognitive interaction *→* emotional interaction *→* behavioral interaction” sequence observed in earlier studies.

Specifically, in video DJY2, cognitive interaction dominated overwhelmingly and increased over time, whereas behavioral and emotional interactions remained low and fluctuated in tandem. In video MCBL1, due to a strong sense of presence and life-like relevance, viewers’ behavioral interactions surged within the first minute. In video YYTLY1 (the last video posted by the senior influencer before his passing), the emotion of “collective mourning” was strongly evoked, making emotional interaction the dominant and continuously intensifying mode throughout the video.

### Sentiment Polarity

**Fig. 6:**
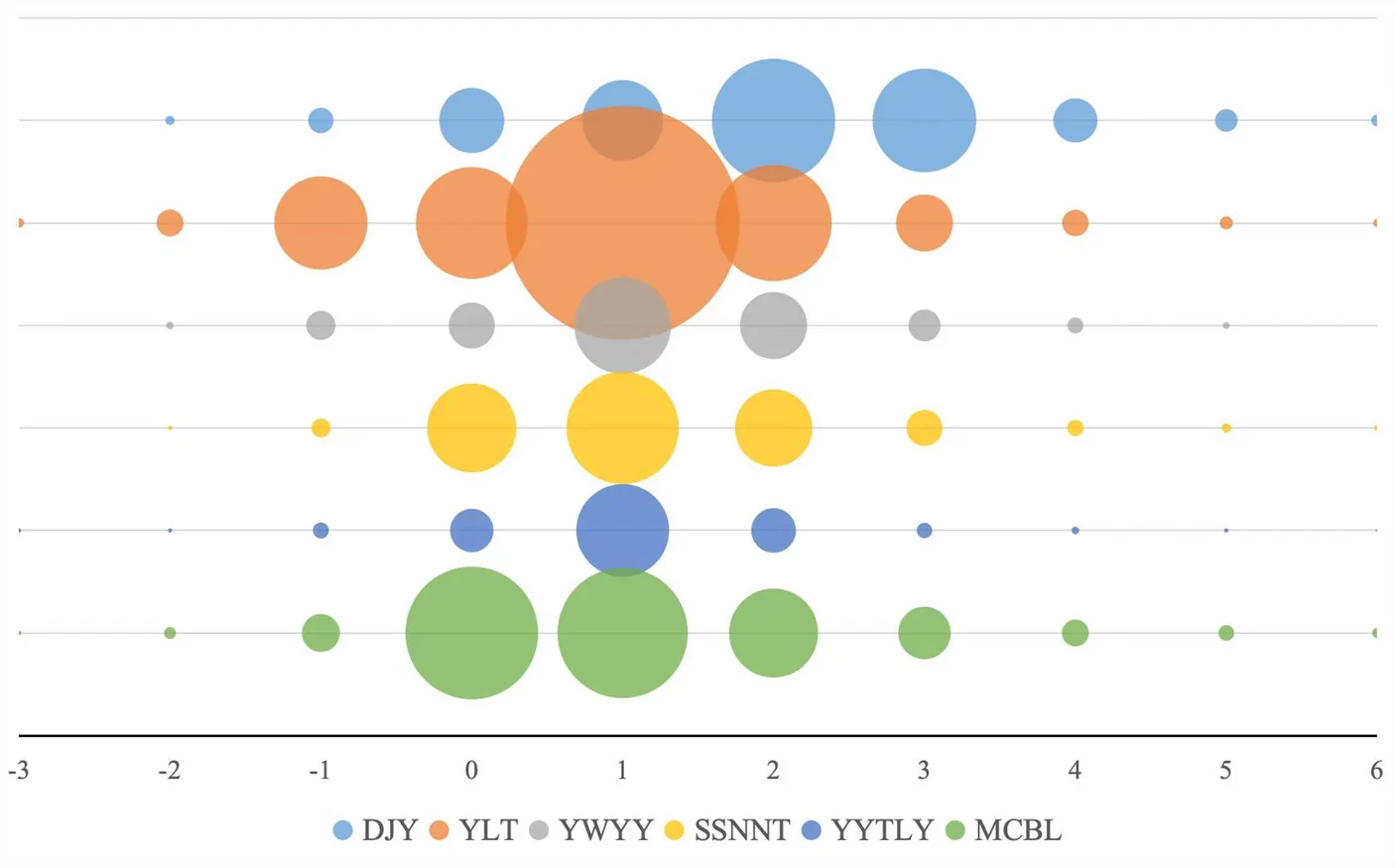
Sentiment polarity distribution map

The sentiment polarity reveals that the danmaku comments for the six SDIs are predominantly characterized by positive emotions (the bubble area corresponding to positive scores is consistently larger than that of negative scores), supplemented by neutral emotions (bubbles with a score of 0), with negative emotional comments accounting for only a small proportion. Positive emotional scores are concentrated between 1 and 3, while negative emotional scores range from -1 to -2. Overall, positive emotional comments not only dominate in quantity but also show a wider score range, spanning from 1 to 10, compared to negative emotional comments, which range from -1 to -5 (Fig. 6).

A one-sample t-test was conducted on the sentiment scores of the six SDIs. The results showed a mean sentiment score of 1.012, with the weighted average score for each individual SDI being positive. The test yielded a statistically significant result *p < 0.001*, confirming an overall positive sentiment orientation.

### Sentiment Type

**Fig. 7:**
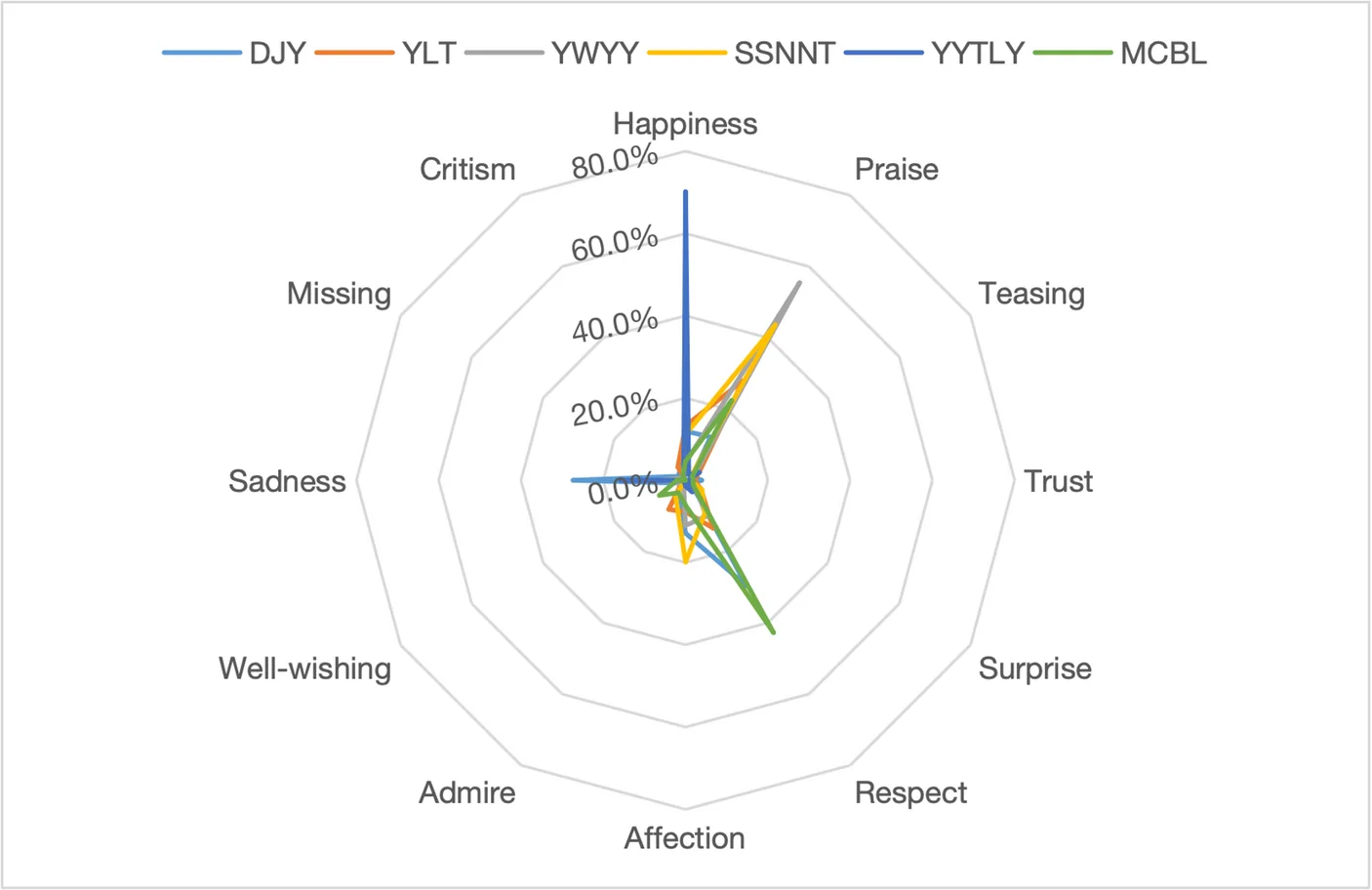
Emotional type chart

Radar charts allow for the inclusion of multiple subjects on a two-dimensional plane for horizontal comparison. Figure 7 illustrates the overall distribution of 12 emotional types, showing a concentration on *praise*, *happiness*, and *respect*, which are all positive sentiment types.^1^

Table 11 illustrates the overall distribution of 12 emotional types, showing a concentration on *praise*, *happiness*, and *respect*. The emotional type of sadness also accounts for a certain proportion. Influencers DJY and YYTLY have experienced the loss of close family members or SDI themselves. They show a higher frequency of sadness-related emotions. YYTLY’s media persona is that of an entertainer, producing humorous and amusing content. Many of his videos even feature playful teasing and pranks from his grandson. In his danmaku, the emotional type of happiness accounts for the highest proportion, while praise and respect are less prominent compared to other SDIs.

**Table 11:** Sentiment distribution across different categories

Sent type	DJY	YLT	YWYY	SSNNT	YYTLY*	MCBL
Positive Sentiment (%)						
Happiness	11.9	13.3	4.3	11.1	70.1	4.7
Praise	12.2	28.0	55.4	43.6	1.5	22.4
Respect	29.6	13.4	10.1	9.3	3.3	42.8
Affection	12.9	7.7	11.0	19.9	1.4	6.1
Admire	3.2	8.2	1.0	4.7	0.5	3.5
Well-wishing	1.4	1.0	1.5	0.5	0.2	7.3
Trust	3.9	2.2	0.7	1.1	0.6	1.9
Surprise	1.6	4.3	2.7	4.6	0.7	2.0
Teasing	1.8	3.8	2.5	1.4	3.9	1.9
Negative Sentiment (%)						
Sadness	27.3	0.6	6.0	0.8	15.7	2.1
Missing	1.9	0.1	1.1	0.1	0.0	0.0
Criticism	2.9	3.8	0.7	1.2	0.9	1.4

## Discussion

**RQ1: What are the defining characteristics of parasocial interaction (PSI) between senior digital influencers (SDIs) and younger audiences in the digital media context?**

### Non-Linear Parasocial Interaction

The temporal analysis of danmaku interactions demonstrates a distinctly nonlinear developmental pattern in parasocial interaction (PSI), challenging the classical view of a progressive, linear sequence from cognition to emotion to behavior (Ge, 2017). Two key findings support this:

First, consider videos centered on profound loss, such as YYTLY’s final video or DJY’s mourning of his wife. In these videos, emotional interaction (collective mourning) emerged dominantly. It preceded cognitive responses like memory recall. This pattern reversed the expected cognitive-to-emotional pathway. Second, in SDI MCBL’s content, cognitive engagement directly stimulated behavioral reactions, such as imitation and motivation. This occurred without the intermediary of heightened emotional involvement. These case-specific trajectories illustrate that cognitive, emotional, and behavioral interactions are not rigidly staged. Instead, they are interconnected and context-responsive. This dynamic, content-driven mode resembles the fluidity of real-life dialogue, where participants naturally shift between different modes of interaction as the conversation progresses.

### The Bidirectional and Socially Embedded Quality of Behavioral Interaction

Beyond its nonlinearity, the PSI observed in this study demonstrates a strong bidirectional quality. This moves beyond the classic unidirectional paradigm of mass media parasocial relationships (Horton & Wohl, 1956). Our findings empirically support and extend the concept of trans-parasocial relations, which are characterized as collectively reciprocal, synchronously interactive, and co-created (Lou, 2022).

In our data, this reciprocal dynamic was most salient in the behavioral dimension, where expressions of *presence and companionship* constituted the dominant form of interaction. The audience’s online companionship translates into tangible emotional support for the SDIs, which in turn influences their media performance. Several examples illustrate this. YYTLY explicitly expressed gratitude and reluctance to part with the audience in his final video before passing (YYTLY1). YWYY recorded a recommended song at the online request of fans (YWYY2). YLT received a game console sent by a fan offline. He responded by recording an “unboxing” video for the “new toy.” This reciprocity suggests that SDIs come to view their audience’s companionship as a vital part of their social reality. It also underscores the significant real-world applicability of these digitally mediated interactions.

### Toward a Deep, Value-Based Parasocial Relation

Our analysis reveals a parasocial relationship between Senior Digital Influencers (SDIs) and younger audiences characterized by notable depth. This depth suggests a connection anchored in shared values and social-emotional resonance, extending beyond superficial exchanges.

Cognitively, interactions were dominated by the audience’s perception and evaluation of SDIs’ content and values. Significant memory recall related to shared human experiences was also observed. This indicates engagement rooted in substantive identification. The audience internalizes the SDIs’ lived experiences and authenticity to sustain a meaningful connection, a process aligning with Kelman’s (1961) conceptualization of identification as a social influence where individuals adopt another’s values and perspectives.

Behaviorally, expressions of *presence and companionship* were overwhelmingly prevalent, followed by those indicating *imitation and motivation*. These supportive behaviors emerged organically across content types without dependence on external incentives, pointing to intrinsically motivated, spontaneous engagement.

Emotionally, interactions featured a high degree of *emotional contagion*, where audiences directly resonated with SDIs’ emotions. This, along with specific instances of *collective mourning*, indicates a dynamic built on shared understanding. It differs from more curated or transactional emotional exchanges. The bidirectional nature of this influence is evident, as seen when SDI YYTLY explicitly acknowledged his audience’s companionship.

In summary, the audience engagement documented here—combining deep cognition, intrinsic behavior, and resonant emotion—outlines a distinct form of parasocial relation. This contrasts with interactions in contexts like live streaming, which are often explicitly gamified and commodified (Li, 2015; Wang, 2019). Instead, it points to a meaningful mode of digital intergenerational bonding based on authenticity, shared values, and a desire for social connection.

**RQ2: How do the technological and communicative features of danmaku platforms shape these PSI characteristics?**

### The Shaping Role of Danmaku Technology: From Collective Mourning to Co-Created Spaces

This section addresses RQ2 by examining how the technological features of the danmaku platform shape the observed characteristics of PSI. Our analysis suggests that danmaku’s unique architecture facilitates a form of PSI that is collectively reciprocal and co-creative, moving beyond the traditional one-sided model (Horton & Wohl, 1956).

A key finding is the prominence of collective mourning in interactions involving SDIs YYTLY and DJY. This phenomenon illustrates how danmaku technology mediates emotional PSI. The platform’s “pseudo-synchronous” feature (Johnson, 2013) is critical here. It allows comments from all viewers to be anchored to specific video timestamps, creating a shared, real-time viewing experience despite physical asynchrony. When YYTLY delivered his farewell message, audiences saw each other’s mourning comments (e.g., “Grandpa, may you rest in peace”) flow in at the same moment in the video. This constructs a powerful sense of co-presence and shared ritual, transforming private grief into a public, collective emotional event.

Furthermore, danmaku’s design supports collective reciprocity and co-creation (Lou, 2022). The anonymity of comments (Chen et al., 2017) may encourage more honest and unrestrained emotional expression, such as raw grief or direct address to the SDI. Visually, these comments overlay the original video, literally co-creating a new textual-visual narrative. The video content and the collective emotional response through danmaku merge into a single, shared artifact. This process fosters what Lou (2022) terms trans-parasocial relations. These are interactions that are synchronously interactive and built collaboratively.

Therefore, danmaku does not merely transmit pre-existing PSI; it actively shapes its form and depth. The technology provides the infrastructure for instantaneous collective feedback and shared emotional rituals. This enables PSI to evolve from a private, imagined relationship into a publicly affirmed, co-created social experience. For SDIs, witnessing this collective response in real-time (e.g., a flood of mourning comments) can make the audience’s presence profoundly tangible, reinforcing the reality and impact of the digital bond. This technologically mediated reciprocity marks a significant shift from the unilateral PSI of the broadcast era.

### Bridging the Intergenerational Divide through Positive Sentiment

Our sentiment analysis reveals a distinct pattern. The parasocial interactions between Senior Digital Influencers (SDIs) and their young audiences lack emotional polarization. Audience feedback is overwhelmingly positive. This consistent positive sentiment is significant for understanding how digital parasocial interaction (PSI) can address the intergenerational divide described in Social Identity Theory.

Social Identity Theory suggests that mere age-based categorization is sufficient to trigger ingroup favoritism and outgroup discrimination (Billig & Tajfel, 1973). Such bias often manifests as prejudice against older adults, including perceptions of incompetence (Fiske et al., 2018). Our data, however, present a contrasting finding. The dominant emotions expressed by the young audience are praise and respect. These positive responses do not match the outgroup discrimination predicted by traditional intergroup theory. Instead, the sustained praise and respect function as affirming social cues. These cues directly counter ageist stereotypes and validate the SDIs’ competence in domains valued by younger people.

This flow of positive feedback may enable an important social psychological shift: re-categorization. For the young audience, the SDI becomes perceived less as a prototypical older adult and more as a skilled creator within a shared interest group. Moving from age-based to skill-based categorization can soften intergenerational boundaries, a process aligned with research on how positive intergroup contact can reduce bias (Chasteen, 2005).

Therefore, these parasocial interactions establish a shared digital space centered on common interests rather than age divisions. Within this space, a young viewer’s appreciation for an SDI’s talent can promote warmer cross-generational understanding. Such an environment actively supports key aspects of active aging. It fosters social integration for older adults across generational lines, strengthens a positive identity beyond age stereotypes, and affirms their valued role within a mixed-age community.

### Positive Evaluations from the Young

Our data show that the parasocial interactions (PSI) in danmaku comments provide specific emotional support to Senior Digital Influencers (SDIs). This support likely contributes to their experience of active aging by moving beyond the role of mere companionship documented in earlier research (Lim & Kim, 2017; Stever, 2008, 2011). Furthermore, it contrasts with studies where PSI may exacerbate distress in contexts of poor offline relationships (Bernhold & Metzger, 2020), highlighting a potentially more beneficial pathway for older adults who are active digital creators.

Critically, this support directly challenges common intergenerational dynamics. While studies often document age-based prejudice from younger toward older adults (Fiske et al., 2018), the emotional feedback from the young audience in our study is overwhelmingly positive, concentrating on praise, happiness, and respect. This creates a constant stream of social recognition that counters ageist stereotypes of incompetence or pity. For example, YYTLY’s humorous content triggered the highest proportion of happiness in comments. This direct, positive link between his creative work and the emotions of a young audience can strengthen his sense of social connection, purpose, and generational value.

Even in cases involving loss, like DJY and YYTLY, the emotion of sadness did not dominate their overall emotional profile. The supportive, positive environment created by the audience remained, suggesting the online community can act as a buffer. This helps SDIs navigate difficult life transitions not through disengagement but through sustained positive social engagement.

In conclusion, danmaku-based PSI creates a distinctive affirming digital mirror for SDIs. It reflects back an image consistent with active aging—being valued, joyful, and respected by a younger generation. This likely nourishes their self-worth, sense of belonging, and motivation to stay engaged, providing key psychological resources that are both cultivated through and contribute to their active participation in the digital sphere.

### Limitations

This study also has several limitations and opportunities for future research. The scope of this study is limited as SDIs represent only a small subset of older adults, with many lacking the digital media skills necessary for such practices. Furthermore, the new media representations of SDIs are likely selective. To integrate into youth-dominated new media platforms, SDIs often portray themselves as healthy, energetic, and aligned with popular youth culture to gain recognition. However, due to the preference of capital for traffic, issues related to aging, such as illness, loneliness, and social isolation, are less likely to gain attention on new media platforms, rendering them invisible. The SDIs who manage to attract significant attention represent only a tiny fraction of China’s vast elderly population, and their selective portrayal may construct an unrealistic image of older adults. Furthermore, this study relies solely on danmaku data and video content for an objective sentiment analysis using sentiment dictionaries, lacking subjective materials. Future research could employ qualitative methods such as interviews and observations to gain deeper insights into the inner worlds of SDIs. This also highlights the need for interventions by national and societal forces in the recommendation and display algorithms of internet platforms to facilitate a more comprehensive and authentic representation of SDIs. Such efforts could promote better intergenerational communication and broader practices of active aging among older adults.

## Conclusion

This study examined the parasocial interactions (PSI) between Senior Digital Influencers (SDIs) and their younger audiences on danmaku video platforms. Through a sentiment analysis of danmaku comments, the research reveals the complex, non-linear nature of these digital relationships and how they are shaped by the unique technological affordances of the danmaku medium.

Our findings demonstrate that these interactions are cognitively substantial, behaviorally supportive, and emotionally resonant. Cognitive engagement, primarily through perception and evaluation, forms the foundation. Behaviorally, expressions of presence and companionship dominate, indicating a relationship built on intrinsic connection rather than transactional exchange. Emotionally, the landscape is overwhelmingly positive, characterized by praise, happiness, and respect, which collectively form a powerful counter-narrative to age-based prejudices.

A key contribution of this study is elucidating how danmaku technology facilitates a shift from traditional, one-sided PSI towards co-creative and reciprocal engagement. The platform’s “pseudo-synchronous” feature fosters shared emotional rituals, such as collective mourning, transforming private sentiment into public, collective experiences. This technologically mediated environment enables what can be understood as value-based parasocial relations, where mutual appreciation grounded in shared interests can help bridge intergenerational divides by shifting categorization from age groups to interest-based affiliations.

In summary, this research highlights that SDIs are not merely subjects of online attention but active agents in redefining digital aging. Their mediated interactions with younger audiences create affirming spaces that support key psychosocial pillars of active aging—social inclusion, positive self-identity, and a sense of purpose. While focused on a digitally adept subset of older adults, this study underscores the transformative potential of interactive platforms in fostering meaningful intergenerational connection. Future research should employ mixed methods to capture subjective experiences and further investigate how digital infrastructures can be designed to support equitable and authentic representation of aging in the digital public sphere.

## Data Availability

The data that support the findings of this study are available from the corresponding author, upon reasonable request.

## Footnotes

1 Note. Based on the sentiment lexicon in the Chinese corpus of Dalian University of Technology, words such as “happiness,” “praise,” and “respect” are considered positive, while “sadness,” “criticism,” and “boredom” are negative. In this study, each positive emotional word is assigned 1 point, and each negative emotional word -1 point. The score increases with the frequency of positive words and decreases with negative ones; when both are equal, the score is 0. In Fig. 6, larger bubbles represent a higher number of danmaku with corresponding emotional scores. , larger bubbles represent a higher number of danmaku with corresponding emotional scores.

## Materials Availability

The materials used in the study are available upon reasonable request from the corresponding author.

## Code Availability

The code used in the study is available from the corresponding author upon reasonable request.
